# Moxifloxacin monotherapy versus combination therapy in patients with severe community-acquired pneumonia evoked ARDS

**DOI:** 10.1186/s12871-017-0376-5

**Published:** 2017-06-14

**Authors:** Tim Rahmel, Sven Asmussen, Jan Karlik, Jörg Steinmann, Michael Adamzik, Jürgen Peters

**Affiliations:** 1 Klinik für Anästhesiologie, Intensivmedizin und Schmerztherapie, Universitätsklinikum Knappschaftskrankenhaus Bochum, In der Schornau 23-25, D-44892 Bochum, Germany; 2 Klinik für Anästhesiologie und Intensivmedizin, Universität Duisburg-Essen and Universitätsklinikum Essen, D-45122 Essen, Germany; 3 Institut für Medizinische Mikrobiologie, Universitätsklinikum Essen, D-45147 Essen, Germany

**Keywords:** Acute respiratory distress syndrome, CAP, 30-day mortality, Liver failure, S3 guideline, Consensus guideline

## Abstract

**Background:**

We tested the hypothesis that moxifloxacin monotherapy is equally effective and safe as a betalactam antibiotic based combination therapy in patients with acute respiratory distress syndrome (ARDS) evoked by severe community acquired pneumonia (CAP).

**Methods:**

In a retrospective chart review study of 229 patients with adult respiratory distress syndrome (ARDS) admitted to our intensive care unit between 2001 and 2011, 169 well-characterized patients were identified to suffer from severe CAP. Patients were treated with moxifloxacin alone, moxifloxacin in combination with ß-lactam antibiotics, or with another antibiotic regimen based on ß-lactam antibiotics, at the discretion of the admitting attending physician. The primary endpoint was 30-day survival. To assess potential drug-induced liver injury, we also analyzed biomarkers of liver cell integrity.

**Results:**

30-day survival (69% overall) did not differ (*p* = 0.89) between moxifloxacin monotherapy (*n* = 42), moxifloxacin combination therapy (*n* = 44), and other antibiotic treatments (*n* = 83). We found significantly greater maximum activity of aspartate transaminase (*p* = 0.048), alanine aminotransferase (*p* = 0.003), and direct bilirubin concentration (*p* = 0.01) in the moxifloxacin treated groups over the first 10–20 days. However, these in-between group differences faded over time, and no differences remained during the last 10 days of observation.

**Conclusions:**

In CAP evoked ARDS, moxifloxacin monotherapy and moxifloxacin combination therapy was not different to a betalactam based antibiotic regimen with respect to 30-day mortality, and temporarily increased markers of liver cell integrity had no apparent clinical impact. Thus, in contrast to the current S3 guidelines, moxifloxacin may also be safe and effective even in patients with severe CAP evoked ARDS while providing coverage of an extended spectrum of severe CAP evoking bacteria. However, further prospective studies are needed for definite recommendations.

## Background

Acute respiratory distress syndrome (ARDS) is a life-threatening disease evoked by a variety of precipitants, including community acquired pneumonia (CAP) with a current mortality of about 40% [1–4]. Severe CAP is the most frequent cause of ARDS [5] and acknowledged recommendations for antimicrobial therapy are provided in Germany by the *S3-guidelines* [6–8] and in the United States by the *Consensus guidelines on the management of CAP in adults* [9]. Along these recommendations, a combination of a broad-spectrum beta-lactam antibiotic (e.g. cefotaxim, ceftriaxone, piperacillin/tazobactam, ertapenem, or meropenem) in combination with a macrolide or fluoroquinolone is usually administered empirically to patients with severe CAP and septic disease progression or mechanically ventilated patients [7, 9, 10]. Fluoroquinolone monotherapy (levofloxacin or moxifloxacin) is reserved for patients without severe disease progression [7, 9, 11]. However, the recommendations to limit moxifloxacin monotherapy to patients with lesser CAP severity is supported only by evidence grade II A and is due to the lack of efficacy data on fluoroquinolones as monotherapy for the treatment of severe CAP [7, 9, 11]. Although, one study reported that an antibiotic combination therapy improved survival among critically ill patients with bacteriaemic pneumococcal illness compared to those treated with beta-lactam monotherapy [12]. Third generation fluoroquinolones were either not used for monotherapy in this study [12] or further studies dedicated to this issue did not exist at that time. However, several studies have suggested sufficient efficacy of fluoroquinolones monotherapy in CAP in the absence of septic disease progression or mechanical ventilation [13–22].

Specifically, moxifloxacin has a broad spectrum of activity against almost all microorganisms isolated from CAP patients and its activity is not opposed by relevant antimicrobial resistance, including multi-resistant pneumococci and pathogens such as *Moraxella catarrhalis* and *Haemophilus influenzae.* Those can carry resistance to penicillins, macrolides, and tetracyclines. Moxifloxacin also possesses the best activity against other pathogens such as *Legionella pneumophila*, *Chlamydophila pneumoniae*, and *Mycoplasma pneumonia*) and is therefore the first choice antibiotic for such infections [23, 24]. Meta-analysis data suggests that moxifloxacin alone has a higher pathogen eradication rate than a β-lactam-based combination therapy [25]. Accordingly, moxifloxacin may be sufficient even when used as a monotherapy in severe CAP. Nevertheless, studies addressing the effectiveness of moxifloxacin monotherapy in severe CAP evoked ARDS are lacking.

Accordingly, we assessed the hypothesis that moxifloxacin monotherapy is equally effective as a guideline recommended combination therapy in ARDS patients due to severe CAP.

## Methods

**Fig. 1:**
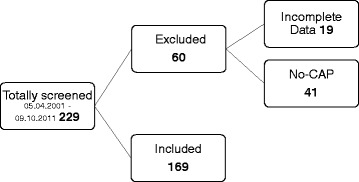
Consort flow chart of inclusion and exclusion of study patients

This retrospective study was reviewed and approved by the Ethics Committee of the Medical Faculty of the University of Duisburg-Essen (no. 052776) and informed consent was obtained from patients or their guardians. We eventually included 169 patients with CAP evoked severe ARDS (98 males (58%), 71 females (42%), mean age: 43 years ±13.8 SD), with the need of antibiotic treatment due to an suspected or proven bacterial infection, selected from 229 patients with ARDS from our intensive care unit (ICU) ARDS database (Fig. 1). Patients with a proven or suspected combined viral infection with bacterial superinfection received antimicrobiotic treatment accordingly (*n* = 41). Forty-one Patients were excluded because ARDS was evoked by trauma or other diseases than CAP and in 19 cases essential data was not documented. Patients with no need of an antibiotic treatment were not included into this study, e.g. patient suffering from solitary viral infection.

**Fig. 2:**
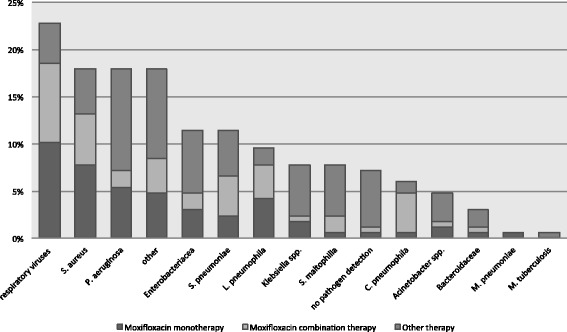
Cumulative frequencies of specific infectious agents detected in respiratory specimen, blood cultures, and/or by serologic and urine antigen tests in the whole cohort of 169 patients during the observation period of 30 days. Bacteria of the normal human flora were not considered

Community-acquired pneumonia was defined as a primary lung infection of bacterial or viral etiology, without history of prior hospitalization [6, 7]. Diagnosis of CAP and classification of severity was accomplished according to guidelines valid at the time of admission [6]. Patients had been admitted to our ICU between 2001 and 2011 and all fulfilled the joint American/European Consensus Committee criteria for ARDS [26]. Patients were treated with a multimodal concept, which included analgosedation, fluid administration, lung-protective mechanical ventilation, as well as hemodynamic, antibiotic, and diagnostic management. Continuous hemofiltration or hemodialysis was performed according to standardized protocols, if required. Microbiologic data were gathered from frequent samples in every patient (e.g., blood cultures, broncho-alveolar lavage, tracheal aspirates, catheter tip cultures). A summary of the pathogen-associated spectrum based on the first detected germ, including the pathogens triggering a secondary or superinfection is shown in Fig. 2.

For study inclusion, a minimum of clinical and demographic data had to be documented, including preexisting morbidities and prior treatments (including antibiotics), Lung Injury Score, Simplified Acute Physiology Score II, Sepsis-related Organ Failure Assessment Score (SOFA), body mass index, necessity for continuous hemofiltration/dialysis, mode and settings of mechanical ventilation, PaO_2_/FiO_2_ ratio (Horowitz Index), establishment of extracorporal membrane oxygenation (ECMO) therapy, results of microbiologic cultures and related modifications of antibiotic therapy, and blood chemistry values. This included markers of liver cell integrity (such as aspartate transaminase: AST; alanine aminotransferase: ALT; glutamate dehydrogenase: GDH), of cholestasis (total bilirubin, direct bilirubin, gamma glutamyltransferase: GGT, alkaline phosphatase: ALP), and those of liver synthetic function (prothrombin time) in order to evaluate the influence of antibiotic therapy on drug-induced liver injury. The observation period was defined from admission to our ICU either to day 30 of hospital stay or death.

### Antibiotic regimens

The patients were allocated to three groups according to the antibiotic regimens they received at the discretion of the ICU attending physician upon admission on day 1 of the observation period. 1) Monotherapy with moxifloxacin intravenously 1 × 400 mg per day (42 patients), 2) combination therapy with moxifloxacin intravenously 1 × 400 mg per day (44 patients) plus a broad-spectrum betalactamase resistant antibiotic (piperacillin, ceftazidime, cefotiam, meropenem, or imipenem), and 3) any other antibiotic regimen based on a broad-spectrum batalactamase resistent antibiotic (see above) and a macrolide (83 patients). Patient’s characteristics and clinical data are depicted in Table 1. Monotherapy was accepted as appropriate in all cases without high risk for multidrug-resistant bacteria. Evaluation of microbiological therapy was followed up on a daily basis for detected microorganisms and on antibiotic susceptibility reports. This was supported by weekly consultations of infectious disease physicians. The duration of antimicrobial therapy was based on subsequently available culture results, clinical recovery, and normalization of laboratory infection values.

**Table 1:** Baseline characteristics and 30-day survival of patients with ARDS for the whole cohort and stratified for in three antibiotic regimen

	Overall cohort	Moxifloxacin monotherapy	Moxifloxacin combination therapy	Other antibiotic therapy	*P*-value
Total number (%)	169 (100)	42 (25)	44 (26)	83 (49)	
Survivors 30-days (%)	117 (69)	28 (67)	31 (70)	58 (70)	0.915
Non-Survivors 30-days (%)	52 (31)	14 (33)	13 (30)	25 (30)	
Characteristics					
Age yrs. (range/± SD)	43 (18–75)	45 (±13.1)	41 (±12.5)	43 (±14.7)	0.473
Male gender (%)	98 (58)	31 (74)	25 (57)	42 (51)	0.045
Female gender (%)	71 (42)	11 (26)	19 (43)	41 (49)	
BMI kg/m^2^ (±SD)	27,65 (±7.3)	30,0 (±8.6)	27,7 (±6.3)	26,4 (±6.7)	0.016
ECMO-Therapy (%)	51 (30)	19 (45)	13 (30)	19 (23)	0.070
Dialysis (%)	83 (49)	25 (59)	22 (50)	36 (43)	0.337
Antibiotics before ICU admission (%)	142 (84)	35 (83)	38 (86)	69 (83)	0.885
Cardiovascular disease (%)	25 (14)	4 (10)	6 (14)	15 (14)	0.432
Prior lung disease (%)	20 (12)	4 (10)	7 (16)	9(18)	0.609
SAPS II (±SD)	46 (±19.5)	47 (±19.2)	40 (±17.7)	49 (±19.5)	0.057
LIS (±SD)	3,2 (±0.56)	3,1 (±0.54)	3,1 (±0.59)	3,3 (±0.56)	0.187
SOFA (±SD)	13 (±6.7)	15 (±7.3)	13 (± 5.6)	12 (±4.4)	0.131
Horowitz-index upon ICU admission (±SD)	152 (±115.7)	135 (±99.5)	221 (±153.0)	124 (±80.4)	0.001

Data are presented as n (%); mean (± SD) or median(range); *BMI* body-mass-index, *ECMO* extracorporeal membrane-oxygenation, *SAPS II* simplified acute physiology score, *LIS* Lung-Injury-Score, *SOFA* Sepsis-related organ failure assessment score; Horowitz-Index: p_a_O_2_/F_i_O_2_; *p*-values were calculated using the Chi-squared test for categorical variables, ANOVA for continuous parametric variables, and the Kruskal-Wallis test for continuous nonparametric variables

### Statistical analyses

Statistical analyses were performed using SPSS (version 21, IBM, Chicago, IL) and power analysis for endpoint survival using G*Power (version 3.1.9.2, Heinrich-Heine-Universität Düsseldorf, Düsseldorf, Germany, http://www.gpower.hhu.de/) revealed a power of 6.3% regarding the number of included patients. Continuous variables are presented as means and standard deviation or expressed as range. All values of variables were grouped according to the patient’s antibiotic treatment regimen. Comparison of values of variables between groups was performed using the Pearson’s chi-squared test for categorical variables, two-way analysis of variance (including Bonferroni’s post hoc test adjustment) for continuous parametric variables, and the Kruskal-Wallis-test (followed by post-hoc Mann-Whitney-U-tests) for continuous non-parametric variables, respectively. Measurements in survivors vs. non-survivors (Table 2) were investigated for differences using a two-sided Student t-test for continuous variables, a Mann-Whitney-U-test for continuous non-parametric variables, and Pearson’s chi-squared test for categorical variables. Differences with an alpha-error *p* of less than 0.05 were regarded as statistically significant.

**Table 2:** Baseline characteristic of survivors and non-survivors (30 days)

	Survivors 30-days *n* = 117 (69)	Non-survivors 30-days *n* = 52 (31)	*P*-value
Characteristics			
Age yrs. (range/± SD)	40,8 (±14.2)	47,2 (±11.7)	0.005
Male gender (%)	65 (56)	33 (63)	0.337
Female gender (%)	52 (44)	19 (37)	
BMI kg/m2 (±SD)	28,1 (±7.7)	26,7 (± 6.2)	0.348
ECMO-Therapy (%)	31 (27)	20 (38)	0.099
Dialysis (%)	49 (42)	34 (65)	0.002
Antibiotics before ICU admission (%)	97 (83)	45 (87)	0.552
Cardiovascular disease (%)	14 (12)	11 (21)	0.120
Prior lung disease (%)	11 (9)	9 (17)	0.142
SAPS II (±SD)	43 (±19.0)	53 (±19.1)	0.003
LIS (±SD)	3,2 (±0.56)	3,2 (±0.56)	0.702
SOFA (±SD)	12 (±5.5)	15 (±5.8)	<0.001
Horowitz-index upon ICU admission (±SD)	158 (±122.5)	140 (±98.5)	0.480

Data are presented as n (%); mean (± SD) or median(range); *BMI* body-mass-index, *ECMO* extracorporeal membrane oxygenation, *SAPS II* simplified acute physiology score, *LIS* lung-injury-score, *SOFA* Sepsis-related organ failure assessment score; Horowitz-Index: paO2/FiO2; *p*-values were calculated using the Chi-squared test for categorical variables, t-test for continuous parametric variables, and the Mann&Whitney U-test test for continuous nonparametric variables

The antibiotic treatment regimens were also compared in relation to 30-day survival using the Kaplan-Meier method with a log-rank test. The impact on 30-day survival of the choice of antibiotic treatment, age, gender, height, weight, pulmonary or cardiac pre-illnesses, ECMO therapy, dialysis or hemofiltration, Simplified Acute Physiology II Score, Lung Injury Score, SOFA Score, and the Horowitz Index as prognostic factors for clinical outcome were analyzed by multivariate Cox regression analysis with stepwise removal of non-significant variables (*p* > 0.05) from the model. Hazard ratios (HR) and 95% confidence intervals (CI) were calculated from the Cox regression analysis to describe the effect of covariates on the hazard.

Biomarkers of liver cell integrity, cholestasis, and liver function were assessed by the Kruskal-Wallis and Monte Carlo tests, comparing the peak values of these markers. In case of a significant result, a time-dependent analysis of the respective data means over three periods (days 0 to 10, 11 to 20, and day 21 to 30, respectively) was performed by the Kruskal-Wallis test to describe a potential time-dependence. Variances in all calculations were regarded as statistically significant within an a priori alpha error *p* of less than 0.05.

## Results

**Fig. 3:**
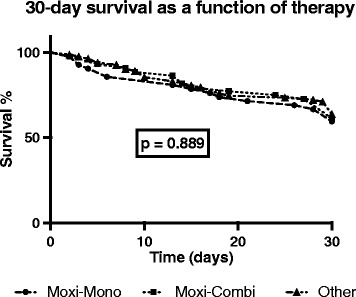
Thirty-day survival in patients with severe CAP evoked acute respiratory distress syndrome (ARDS) stratified for the antibiotic treatment regime after ICU admission. Kaplan-Meier estimates were used to calculate probabilities of 30-day survival. Thirty-day survival was not significantly different (*p* = 0.889) between moxifloxacin monotherapy (Moxi-Mono), moxifloxacin combination therapy (Moxi-Combi), and other regimens (Other)

30-day survival (67% overall) did not differ (*p* = 0.89, Fig. 3) between the antibiotic regimen groups, with a statistical power of 6.3%. Fourteen patients (33%) died in the moxifloxacin monotherapy group, 13 patients (30%) in the moxifloxacin combination therapy group, and 25 patients (30%) in the group treated with other antibiotics.

The most common cause of CAP evoked severe ARDS were bacterial infections (*n* = 115, 68%), followed by viral infections (*n* = 41; 24%), 68% (*n* = 28) of the latter caused by *Influenza-A (H1N1)*. No specific pathogen was detected in 8% (*n* = 13) of all patients. Most frequently detected bacteria were *S. aureus* (23%, *n* = 39), *P. aeruginosa* (11%, *n* = 19), *S. pneumoniae* (11%, *n* = 19), *Enterobacteriacea* (11%, *n* = 18), and *L. pneumophila* (10%, *n* = 17). In almost all patients moxifloxacin showed a suitable spectrum of antimicrobial activity against the initially detected bacterium. A summary of the pathogen-associated spectrum, based on the first detected germ and also including pathogens presumably triggering a secondary or superinfection, is shown in Fig. 2.

Looking at the antibiotic pretreatment the regimens were distributed equally between the three groups. In detail, 2 patients in the moxifloxacin-monotherapy group were pretreated with moxifloxacin alone, 6 patients were pretreated with moxifloxacin in combination with a ß-lactam antibiotic, 27 received other ß-lactam based regimen without using moxifloxacin and 7 patients were not treated with antibiotics till admission on our ICU. In the moxifloxacin combination-group 2 patients were pretreated with moxifloxacin alone, 3 in combination with beta-lactam antibiotics, 33 patients without using moxifloxacin and 6 patients did not receive any antibiotics till admission. In the other treatment group one patient received moxifloxacin alone and 2 patients received moxifloxacin in combination, 65 were pretreated with a regimen not using moxifloxacin and 14 patients were not given antibiotics until admission. Therefore, respective antibiotic treatments had been started in 84% of all patients prior to ICU admission, with no significant impact on 30-day survival (*p* = 0.42) compared to patients without antibiotic pretreatment.

Comparing mean duration of ICU stay, we found the shortest duration in the moxifloxacin-monotherapy group (24.6 days ±18.15) compared with the moxifloxacin combination therapy (25.1 days ±19.28), and the control group (27.6 days ±25.83), albeit without reaching significance (*p* = 0.797). Furthermore, there was no significant difference in the frequency of antibiotic changes between the three groups over the 30-day observation period (*p* = 0.805).

Extracorporeal gas exchange for severe lung failure was necessary in 51 of 169 patients (30%), and hemodialysis/hemofiltration was established in 83 patients (49%) within the 30-day observation period. Overall duration of stay in the ICU averaged 26 days ±22.4. Patient characteristics are illustrated in Tables 1 and 2.

Age (*p* = 0.473), ECMO therapy (*p* = 0.07), dialysis (*p* = 0.337), antibiotic treatment before ICU admission (*p* = 0.89), pulmonary (*p* = 0.61) or cardiac disease (*p* = 0.43), SOFA Score (*p* = 0.13), Lung Injury Score (*p* = 0.19), and the SAPS II Score (*p* = 0.057) were distributed equally across groups.

We found some differences regarding the demographic characteristics between groups (Table 1), i.e., gender (*p* = 0.045), body mass index (*p* = 0.016), and Horowitz Index (*p* = 0.001). Body mass index was 13.6% greater (*p* = 0.006) and there were 23% more males in the moxifloxacin monotherapy group compared to other groups (Table 1). Furthermore, the Horowitz Index was 38.9% less in the moxifloxacin monotherapy group compared to the moxifloxacin combination therapy group (*p* < 0.001, Table 1). The Horowitz Index did not differ between the moxifloxacin monotherapy group and the combination therapy group (*p* = 0.59).

Multivariate proportional hazard analysis by Cox regression revealed age (hazard ratio (HR): 1.03; 95% confidence interval (CI) 1.0–1.05), *p* = 0.029), body mass index (HR 0.95; 95% CI 0.89–1.00), *p* = 0.042), cardiac preconditions (HR 2.2 (95% CI 1.1–4.4), *p* = 0.026) and hemodialysis/hemofiltration (HR 2.5; 95% CI 1.3–4.8), *p* = 0.004) as independent risk factors for 30-day survival (Table 3). Thus, ARDS patients without cardiac preconditions showed a more than twofold greater 30-day survival compared to patients with preexisting cardiac disease and patients needing hemodialysis or hemofiltration showed a 2.5 greater mortality (Table 3).

**Table 3:** Multivariate stepwise Cox regression analysis with Hazard ratio

	*p*-value	HR (95% CI)
Antibiotic regime		
Other antibiotic therapy		
Moxifloxacin monotherapy	0.86	
Moxifloxacin combination-therapy	0.99	
Sex	0.653	
Age	0.029	1.03 (1.00–1.05)
BMI	0.042	0.95 (0.90–1.00)
History of prior pulmonary disease	0.481	
History of prior cardiovascular disease	0.027	2.12 (1.06–4.35)
ECMO-Therapy	0.222	
Dialysis	0.005	2.48 (1.31–4.71)
Horowitz-Index upon ICU admission	0.987	
SAPS-II	0.313	
LIS	0.477	
SOFA-Score	0.358	

*HR* hazard ratio (specified in case of significant *p*-value of multivariate stepwise Cox regression analysis), *CI* confidence interval, *BMI* body-mass-index, *ECMO* extracorporeal membrane Oxygenation, SPAS: simplified acute physiology score, *LIS* Lung-Injury-Score, *SOFA* Sepsis-related organ failure assessment; Horowitz-Index: pO_2_/F_i_O_2_

**Fig. 4:**
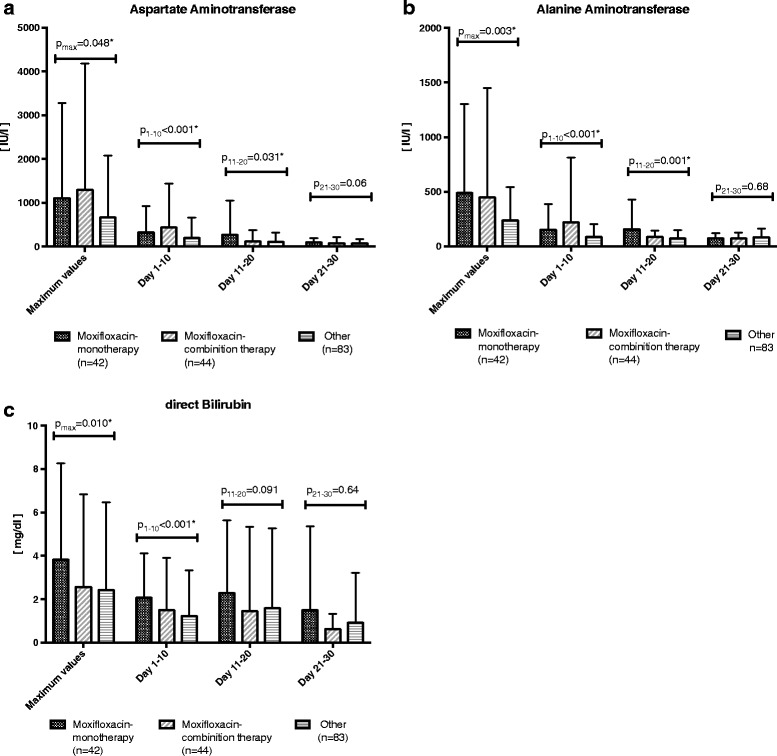
Time course of liver function markers in patients with ARDS as stratified by antibiotic regime after admission to ICU. Group means are depicted as a bar chart with corresponding standard deviation. **a** Aspartate aminotransferase activity. The maximum values of each patient (p_max_ = 0.048) and the average values of the first 10 days (p_1–10_ < 0.001) and from days 11–20 (p_11–20_ = 0.031) were significantly different between moxifloxacin monotherapy (Moxi-Mono), moxifloxacin combination therapy (Moxi-Combi), and other regimens (Other). In, there was no significant difference between during the last 10 days (p_21–30_ = 0.06). **b** Alanine aminotransferase activity. The maximum values of each patient (p_max_ = 0.003) and the average values in the first 10 days (p_1–10_ < 0.001) and from day 11–20 (p_11–20_ = 0.001) were significantly different between moxifloxacin monotherapy, moxifloxacin combination therapy (Moxi-Combi), and other regimens (Other). In contrast, there was no significant difference in the last 10 days (p_21–30_ = 0.68). **c** Direct bilirubin concentration. The maximum values of each patient (p_max_ = 0.010) and the average values in the first 10 days (p_1–10_ < 0.001) were significantly different between moxifloxacin monotherapy (Moxi-Mono), moxifloxacin combination therapy (Moxi-Combi), and other regimens (Other). In contrast, there was no significant difference from day 11–20 (p_11–20_ = 0.091) and in the last 10 days (p_21–30_ = 0.64)

Liver tests were abnormal in all groups and there were significant differences between groups when comparing maximum AST activity (*p* = 0.048), ALT activity (*p* = 0.03), and direct bilirubin concentration (*p* = 0.01). When moxifloxacin was used within the first 10 days (both in monotherapy and combination therapy groups), a 65–123% increased AST activity (*p* < 0.001, Fig. 4), a 81–16% greater ALT activity (*p* < 0.001, Fig. 4), and a 23–69% greater direct bilirubin concentration (*p* < 0.001, Fig. 4) was seen compared to the regimens not using moxifloxacin. However, initially abnormal liver function variables diminished over time and between group differences faded over time. Accordingly, during days 21–30 we found no significant differences for AST activity (*p* = 0.06), ALT activity (*p* = 0.68), or direct bilirubin concentration (*p* = 0.64) under moxifloxacin regimens when compared to the non-moxifloxacin group (Fig. 4). Other liver related tests (GDH, GLDH, GGT, ALP, prothrombin time) showed no significant differences between groups.

## Discussion

This retrospective study shows, that moxifloxacin monotherapy is effective and not associated with excess 30-day mortality in patients with severe CAP evoking ARDS (Fig. 3). Furthermore, initially and temporarily increased liver function markers had no apparent influence on outcome and elevated values normalized over time. Therefore, moxifloxacin monotherapy may be an effective and safe antibiotic treatment even in CAP evoked severe ARDS while extending the spectrum of susceptible bacteria.

Our findings supplement the recommendations of both the current German and American guidelines, which prefer beta-lactam antibiotic based combination therapies [6, 7, 9] over moxifloxacin monotherapy. However, other studies have also reported that moxifloxacin monotherapy can be as effective and safe as a combination regimen in patients with severe CAP and is not associated with relevant disadvantages for the patient’s recovery [18, 20–22]. Furthermore, Vardakas et al. [21] showed in a meta-analysis that the use of respiratory fluoroquinolones was associated with higher treatment success and a better clinical outcome when compared to the combination therapy of a ß-lactam antibiotic and a macrolide. In particular, treatment success as defined by resolution of two or more baseline symptoms or signs of infection, especially in cases with more severe CAP, was significantly higher among patients who received fluoroquinolones. While we could not demonstrate superiority of moxifloxacin monotherapy, even showing no difference is an important finding since using one drug instead of multiple antibiotics might be beneficial alone by reducing costs and, potentially, development of antibiotic resistance in other ICU patients. Comparing duration of ICU stay our data correspond well with the above mentioned studies. In addition, the shortest duration was found in the moxifloxacin-monotherapy group (24.6 days) compared to the control group (27.6 days), albeit without showing statistical difference. Additionally, our data showed no significant different frequency of antibiotic-changes between the three groups.

Moxifloxacin covers most bacteria evoking CAP and is more effective against of the whole spectrum of CAP related pathogens compared to beta-lactam antibiotic based combination therapies [9, 18, 19]. A better microbiological effect and lower risk of treatment failures of moxifloxacin [19, 27] can be attributed to its excellent tissue penetration, high bioavailability, a bactericidal mechanism, an effect even on otherwise resistant pathogens, and high effectiveness against pathogens of atypical pneumonia, such as Chlamydia, Legionella, or Mycoplasma. Therefore moxifloxacin is the first choice in pneumonia cases caused by Legionella. Our germ spectrum showed in almost 10% of the cases an infection with Legionella causing severe CAP and ARDS (Fig. 2). Thus, we would submit that a third generation fluoroquinolone should be part of an initially used antibiotic regimen in CAP evoked ARDS, at least unless proper culture results and resistograms have been obtained.

Our data suggest that moxifloxacin monotherapy may be as effective as a betalactam antibiotic based combination therapy with or without fluoroquinolones. In fact, non-difference of antibiotic monotherapy might be evidence that besides the selection of the initial antibiotic and general supportive treatment, outcome likely depends more on the appropriateness of the hosts’ immune response than the antibiotic regime chosen.

### Limitations

Although all ARDS patients were treated with a standardized multimodal concept, we cannot exclude that unknown and potentially confounding factors exist. In fact, we found a somewhat unequal distribution in some variables such as the Body-Mass-Index, gender, and the initial Horowitz Index but well-known independent risk factors for survival, such as cardiac preconditions, hemodialysis/−filtration, or age were not differently distributed between the therapy groups. In fact, a worse gas exchange in the moxifloxacin group, as suggested by a lesser Horowitz index, is a conservative error with respect to the non-difference hypothesis and did not turn out as independent risk factor both in our Cox regression analysis and in previous studies [28]. The spectrum of cultured infectious agents demonstrates for the large part the pathogens typical of community acquired pneumonia in ARDS patients, but their incidence did not show the classic CAP profile. One explanation might be that we examined a peculiar cohort with only the severest CAP evoking ARDS and that such a cohort has a slightly different pathogen spectrum. Another factor might be that 84% of patients had already received an antibiotic treatment before ICU admission, i.e., before we bronchoscopically sampled specimen or collected blood for cultures and therefore the targeted microbiological detection is much more imprecise and difficult, which may have influenced our detected microbiologic spectrum significantly. Additional the exact time and indication as well as the position of the gained samples are not reconstructible based on our recorded facts in our ARDS sheet. Another limitation is the inclusion of viral patients with the need of antibiotic therapy due to suspected or proven bacterial superinfection or co-infection. We did not exclude these patients since the empirical antimicrobial treatment was applicated, this special cohort my have additionally confounding impact on our result. Finally, although our data were derived from a cohort of patients with severe ARDS, our analysis is retrospective and associated with the substantial ß-error due to an underpowered sample size. Accordingly, we cannot exclude that a significant differencemay still be present between groups with a much larger sample size. Furthermore, our exploratory analysis revealed some differential effects on liver enzyme activities in the early observation period when using moxifloxacin which merits further analysis.

## Conclusions

In summary, our retrospective data in a large cohort of ARDS patients allows the conclusion, that moxifloxacin monotherapy may also be an effective and safe empirical treatment option in patients with ARDS caused by severe CAP, with the advantage of covering the largest spectrum of severe CAP evoking infectious agents. Clearly, prospective, randomized controlled trials are needed to further investigate the effects of moxifloxacin monotherapy in patients with ARDS evoked by community-acquired pneumonia to provide definite recommendations.

## Abbreviations

ALP: Alkaline phosphatase
ALT: Alanine aminotransferase
ARDS: Acute respiratory distress syndrome
AST: Aspartate aminotransferase
CAP: Community acquired pneumonia
CI: Confidence interval
ECMO: Extracorporal membrane oxygenation
FiO2: Inspired fraction of oxygen
GDH: Glutamate dehydrogenase
GGT: Gamma-glutamyl transferase
HR: Hazard ratio
ICU: Intensive care unit
PaO2: Arterial partial pressure of oxygen
SAPS II: Simplified Acute Physiology Score II
SOFA: Sepsis-related organ failure assessment score
